# Seasonal and Regional Effects on the Yield and Bioactive Constituents of *Torreya nucifera* Essential Oils in South Korea

**DOI:** 10.3390/plants14213370

**Published:** 2025-11-04

**Authors:** Chanjoo Park, Nahyun Kim, Mi-Jin Park

**Affiliations:** Forest Industrial Materials Division, Forest Products and Industry Department, National Institute of Forest Science, Seoul 02455, Republic of Korea; chanjoopark515@korea.kr (C.P.); knh1125@korea.kr (N.K.)

**Keywords:** *Torreya nucifera*, essential oils, 3-carene, *D*-limonene, season, region

## Abstract

The essential oil of *Torreya nucifera*, a coniferous tree native to East Asia, has notable bioactive properties with potential industrial applications. This study examined the effects of seasonal and regional factors on the yield and bioactive constituents of *T. nucifera* oils in South Korea. Leaf samples were collected in spring (March), summer (June), and autumn (September) of 2023–2024 from three ecologically distinct regions: Jeju Island, Jinju, and Hwasun. Oil yield was stable across seasons (0.9–1.6%) but varied significantly by region (*p* < 0.05), with Hwasun showing the highest yield. This stability across seasons may reflect the perennial evergreen nature of *T. nucifera*. Gas Chromatography-Mass Spectrometry (GC–MS) identified 32 volatile components, predominantly monoterpenes (83.7–90.4%) and sesquiterpenes (5.4–11.7%), with *D*-limonene and 3-carene as key chemical markers. Notably, 3-carene levels were significantly affected by region (*p* < 0.0001), with higher concentrations in Jeju oils, while *D*-limonene was influenced by season, region, and their interaction (*p* < 0.001), reaching peak levels in Hwasun during summer and autumn (up to 70%). Therefore, *T. nucifera* oil from Hwasun harvested in autumn can be optimised for commercial production by maximising oil yield and enhancing chemical markers.

## 1. Introduction

*Torreya nucifera* Siebold & Zucc., native to Southern Korea, is an evergreen tree belonging to the Taxaceae family. In South Korea, *T. nucifera* is predominantly distributed across the warm temperate regions of the south coastal areas and Jeju Island, where it is primarily conserved as remnant forest stands in the vicinity of Buddhist temples and Confucian shrines [1]. *T. nucifera* grows to 15–25 m tall with a trunk up to 1.5 m in diameter. The leaves are evergreen, needle-like, 2–3 cm long and 3 mm broad with a sharply spiny tip and two whitish stomatal bands on the underside [2]. The essential oils of *T. nucifera* are extracted from various parts, such as seeds, wood, and leaves. In particular, *T. nucifera* seed oil has demonstrated lipid metabolism-improving [3] and anti- diabetic [4] effects. Recently, our research team demonstrated that *T. nucifera* leaf essential oils possess anti-asthma effects, mainly due to *D*-limonene and 3-carene [5]. Furthermore, previous studies by Kim et al. [6] have also reported the therapeutic potential of 3-carene for asthma. The major chemical compounds in the leaf essential oils extracted from *T. nucifera* were *D*-limonene (13.5%), followed by δ-cadinene (10.5%) and α-bisabolol (10.2%) [7]. The leaves of *T. nucifera*, regarded as unused forest biomass, represent a potential bioresource for native essential oil production, offering benefits to both the forestry and essential oil industries through sustainable production. Especially, the bioactive components *D*-limonene and 3-carene in leaf oils hold considerable industrial importance. *D*-limonene, abundant in citrus oils, is valued as a fragrance and ingredient in pharmaceutical, food, and cosmetic products due to its biological activities [8]. 3-Carene is an effective natural inhibitor of food-borne pathogens, suggesting its potential application in the food industry [9].

Due to various intrinsic and extrinsic factors, essential oils exhibit a high variability in their composition, both qualitatively and quantitatively [10]. Among intrinsic factors, chemotypic diversity represents genetic variation within the same species, reflecting plant adaptation to local ecological conditions [11]. For instance, *Cryptomeria japonica* leaf oil shows three chemotypes based on major constituents: (1) ent-kaurene (Taiwan and Korea), (2) elemol + ent-kaurene (Japan, China, and Nepal), and (3) α-pinene (Azores, Corsica, and Japan). In contrast, ecophysiological variations driven by regional and climatic conditions are extrinsic factors that significantly affect the chemical profiles and the yield of oils [12]. Such regional differences have also been reported for *Myrtus communis* L., with notable variations in myrtle oils composition among populations from Turkey [13], Italy [14], and Iran [15]. Another factor influencing essential oils is seasonality, which affects their biosynthesis, storage, and emission, particularly with reproductive and vegetative life cycles as well as stress conditions [16]. Additionally, diurnal cycles can alter the accumulation of bioactive compounds through fluctuations in enzymatic activity, mainly driven by changes in temperature and light conditions [17]. Consequently, seasonal variation in essential oils has been investigated in commercially important crops, including rosemary (*Rosmarinaus officinalis*) [18], tea tree (*Melaleuca alternifolia*) [19], dill (*Anethum graveolens* L.), and parsley (*Petroselinum crispum* (Mill.) Fuss) [20]. Similarly, comparing the *T. nucifera* oils across regions and seasons can help optimise strategies for maximising targeted bioactive compounds [16,21]. Hence, harvest timing of aromatic crops is crucial, as it can influence both the yield and chemical profile depending on species and environmental conditions [22,23]. To date, no studies have investigated the effects of seasonal and geographic variation *on T. nucifera* oils in South Korea.

Therefore, this study investigates the influence of seasonal and regional factors on the yield and bioactive constituents (3-carene and *D*-limonene) of leaf oils extracted from *T. nucifera* across South Korea. Specifically, essential oils were extracted via hydrodistillation from leaves collected during spring (March), summer (June), and autumn (September) of 2023–2024 in three ecologically distinct regions: Jeju Island, Jinju, and Hwasun. These findings provide insights into establishing the production of high-quality *T. nucifera* oils enriched in bioactive compounds for potential anti-asthmatic applications.

## 2. Results

### 2.1. The Aroma Characteristics of Leaf Essential Oil Extracted from T. nucifera

**Figure 1:**
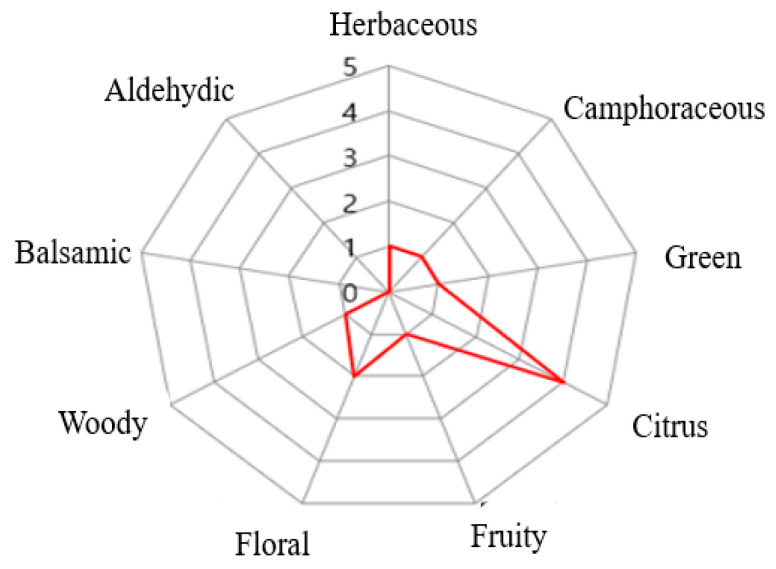
The odour profiles of *T. nucifera* essential oils. The intensity of each characteristic aroma was represented by the red colour.

The fragrance of *T. nucifera* essential oils was characterised by a dominant green citrus note reminiscent of yuzu (*Citrus junos* Tanaka), yet notably lacking in sweetness. As shown in Figure 1, *T. nucifera* essential oils had a stronger intensity of citrus note, whereas fruity, balsamic, and aldehydic notes were absent.

### 2.2. Seasonal and Regional Variations in the Oil Yield of T. nucifera

The oil yield of *T. nucifera* was constant regardless of seasons (0.9 ± 0.2–1.6 ± 0.3% DW), although there was a significant effect (*p* < 0.05) on the yield based on region (Table 1). Furthermore, there was no interaction between season and region affecting the oil yield of *T. nucifera.* The essential oil of *T. nucifera* from Hwasun had a significantly higher oil yield compared to those from Jeju and Jinju. In autumn 2023, the essential oil yield from Hwasun (1.4 ± 0.3%) was significantly higher than that from Jeju (0.9 ± 0.2%), representing an increase of more than 50%.

### 2.3. Effect of Season and Region on Targeted Bioactive Constituents (3-Carene and D-Limonene) in T. nucifera Essential Oil

GC-MS analysis revealed distinct chemical profiles of *T. nucifera* essential oils collected from three regions: Jeju, Jinju, and Hwasun in spring 2023 (Table 2). A total of 32 volatile compounds were identified, accounting for 92.8–95.9% of the total oils. The *T. nucifera* oils consisted mainly of monoterpenes (83.7–90.4%) and sesquiterpenes (5.4–11.7%). Across all regions, the components of α-pinene, 3-carene, and *D*-limonene were the predominant constituents, representing 79.4–86.5% of the oils. In particular, α-pinene was abundant in all samples, accounting for 22.61% to 32.96% of total oils.

In Jeju oils, 3-carene (26.6 ± 5.2%) and *D*-limonene (37.4 ± 8.4%) occurred at similar levels, whereas in Jinju and Hwasun oils, *D*-limonene (40.9 ± 5.9% and 36.8 ± 5.8% respectively) was more markedly abundant than 3-carene (12.6 ± 3.3% and 11.6 ± 3.1%, respectively). *T. nucifera* essential oils from Jinju contained higher levels of sesquiterpenes compared to those from Jeju and Hwasun. Notably, the Jinju oil was characterised by a higher content of sesquiterpenes hydrocarbons (7.7 ± 1.0%), which was approximately twice the level found in Hwasun oils (3.5 ± 0.8%) and nearly five times higher than that in Jeju oils (1.4 ± 0.1%). In contrast, the levels of oxygenated sesquiterpenes in oils were relatively consistent across all regions, ranging from 3.2–4.0%. Based on chemical profiling of *T. nucifera* essential oils from different regions (Table 2), the chemical components of 3-carene and *D*-limonene were selected as chemical markers for quality control.

**Figure 2:**
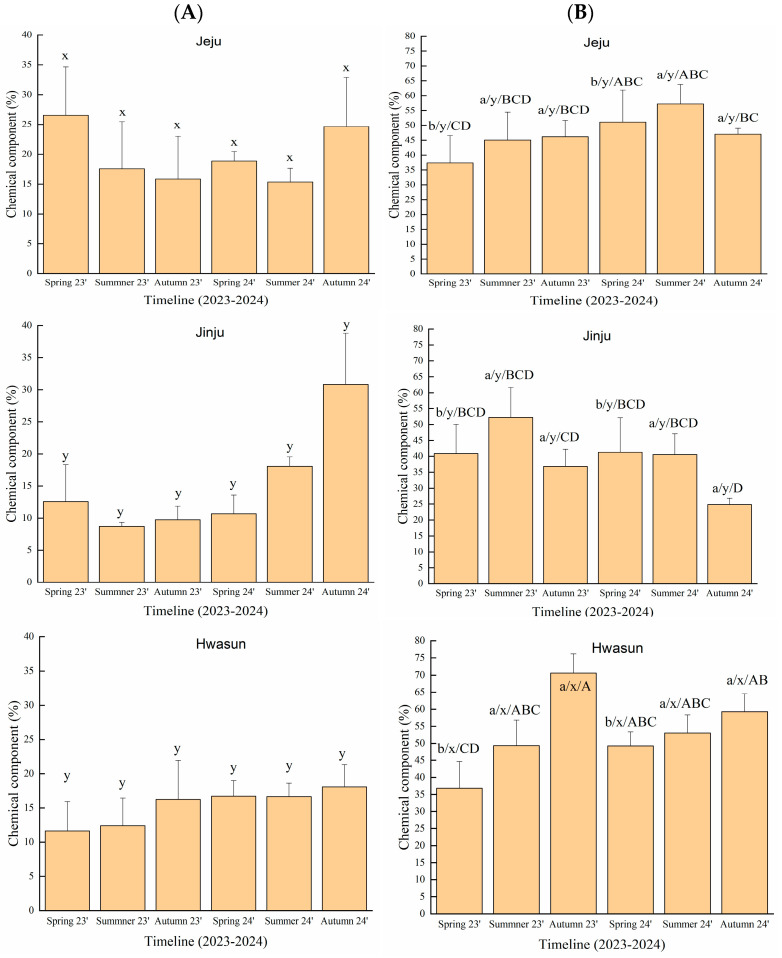
The significant interaction effect and main effect of season and region, respectively, on the targeted chemical constituents (%) of 3-carene (**A**) and *D*-limonene (**B**) in *T. nucifera* essential oil. Values are presented as means ± SD (*n* = 4). Mean values designated by a different letter are significantly different between groups according to a two-way ANOVA test with season and region as variability factors: season for a, b, c and region x, y using lowercase letters, and season × region interaction for capital letters.

Table 3 and Figure 2 present the two-way analysis of variance (ANOVA) results assessing the effects of season, region, and their interaction (season × region) on 3-carene and *D*-limonene concentrations in *T. nucifera* oils.

For 3-carene, region had a highly significant effect, while season and the interaction (season × region) were not significant. With respect to regional variation, essential oils from Jeju exhibited higher 3-carene levels in spring (26.6 ± 8.1%) and summer (17.6 ± 7.9%) compared to those from Jinju (12.6 ± 5.7% and 8.7 ± 0.7%, respectively) in 2023. However, the 3-carene content remained relatively consistent across seasons during the study period, ranging from 8.7–30.8%.

For *D*-limonene, significant effects were observed for season, region, and their interaction. Regarding seasonal variation, *D*-limonene concentrations were significantly lower in spring (36.8 ± 7.8% to 51.1 ± 10.8%) than in summer and autumn (40.5 ± 1.5% to 70.6 ± 5.7%) during 2023–2024. In terms of regional differences, essential oils from Hwasun exhibited significantly higher *D*-limonene levels than those from Jinju and Jeju. For instance, the *D*-limonene content in Hwasun oil reached 70.6 ± 5.5%, nearly twice that of Jinju oil (36.8 ± 8.3%) in autumn 2023. Considering the interaction between season and region, *D*-limonene concentrations in oils extracted from Hwasun (36.8 ± 7.8% to 70.6 ± 5.5%) were significantly higher than those from the Jinju regions (24.9 ± 8.7% to 52.2 ± 3.9%) throughout 2023–2024. Lastly, with respect to seasonal factors, there was no significant difference in the chemical markers 3-carene and *D*-limonene between 2023 and 2024 (Figure 2).

Additionally, principal component analysis (PCA) was performed to examine the relationships among monthly temperature (°C), monthly rainfall and targeted chemical markers (3-carene and *D*-limonene, respectively) (Table 4).

As shown in Table 4, the correlation matrix revealed weak positive correlations between 3-carene and both temperature and rainfall compared with limonene. For *D*-limonene, rainfall (*r* = 0.3107) and temperature (*r* = 0.2125) were the major correlated factors, accounting for approximately 50% of the total variation.

## 3. Discussion

### 3.1. The Effect of Season and Region on the Oil Yield of T. nucifera

The oil yield of *T. nucifera* remained constant regardless of the seasons; however, there was a significant effect on the yield based on region. Moreover, no interaction between season and region affected the yield of *T. nucifera* oils (Table 1). The essential oil of *T. nucifera* from Hwasun exhibited a significantly higher oil yield than those from Jeju and Jinju. This trend is likely attributable to the favourable environmental conditions in the Hwasun region, which promote the production of secondary metabolites, resulting in a higher yield compared to other regions. Similarly, two studies by Jnanesha et al. [24] and Khaiper et al. [25] reported that higher essential oil yields in certain regions were associated with environmental factors that enhance the biosynthesis of secondary metabolites. Jeong et al. [26] investigated conservation management strategies for *T. nucifera* forests. In the Naju region, soil pH ranged from 4.65 to 6.57 (average 5.37), total nitrogen from 0.40% to 1.88% (average 1.02%), and available phosphorus from 15.81 to 22.68 mg/kg (average 18.17 mg/kg). Additionally, *T. nucifera* grows in sandy, loamy, and clay soils with mildly acidic to mildly alkaline pH. It tolerates full or partial shade and prefers moist conditions [27]. Further research is required to determine the favorable environmental conditions in Hwasun through soil analysis. Most importantly, the oil yield and chemical constituents of essential oils are influenced by a combination of factors, including plant genetics, climate, soil conditions, elevation, and the interaction of these elements [28,29,30]. The weather in Hwasun and Jinju, both inland areas, is similar. In contrast, Jeju, being an island, has a unique climate characterised by higher rainfall and temperatures compared to the other provinces. Although there were regional variations across the three provinces, *T. nucifera* essential oils showed a consistent oil yield throughout different seasons. This stability in oil yield may be due to the relatively constant ecological pressures experienced by this species, which is a perennial and evergreen tree [31]. For the potential commercialisation of essential oils, the production process must align with conditions that ensure good process yield and high quality [32]. Conventional methods of essential oil extraction generally result in lower yields and efficiency, as well as longer extraction times, compared to non-conventional techniques such as microwave-assisted and ultrasonic-assisted extraction [33]. Further research is needed to optimise the extraction method of *T. nucifera* essential oils, improving both yield and quality for sustainable industrial use.

### 3.2. The Overall Chemical Profile of T. nucifera Essential Oils

To select the chemical markers of *T. nucifera* oils, GC-MS analysis identified 32 volatile components in the essential oil, accounting for 92.8–95.9% of the total oils (Table 2). Across all collection sites, the major components of *T. nucifera* oils were α-pinene, 3-carene, and *D*-limonene, which together accounted for 79.4–86.5% of the total oils. These results are consistent with a previous study by Yoon, Kim, Oh, Lee and Hyun [2], which reported that *T. nucifera* essential oils from Jeju contained *D*-limonene (30.1%), 3-carene (15.4%), and α-pinene (11.5%) as the predominant components. The three essential oils of *T. nucifera* showed broadly similar chemical profiles; however, notable chemical variations in oils were observed based on the geographic origin. Sharafzadeh and Zare [34] mentioned that some environmental factors can influence biochemical pathways and physiological processes that alter plant metabolism. Also, research on *T. nucifera* essential oils from various regions across South Korea remains limited. Most existing studies have focused solely on samples from Jeju Island. This may be due to the high population density of *T. nucifera* on Jeju, which likely facilitates easier sample collection compared to other regions.

### 3.3. Effect of Region and Season on Chemical Markers of T. nucifera Essential Oil

Following the screening of the chemical profile of *T. nucifera* oils, *D*-limonene and 3-carene were designated as bioactive components and selected as chemical markers for quality control. Li et al. [35] noted that ideal chemical markers should be the therapeutic components of herbal medicines as their quantity can serve as an indicator of herbal medicine quality throughout the production process, covering species authentication, harvesting, product evaluation, and toxic compound detection. Several studies have reported that *T. nucifera* oil exhibits antibacterial and anti-inflammatory activities. These biological effects of *T. nucifera* oil are likely due to bioactive compounds such as *D*-limonene and 3-carene [2]. Hence, *D*-limonene was selected as the primary chemical marker of *T. nucifera* oils. *D*-limonene is a monoterpenoid compound widely present in the citrus oils. It holds significant commercial value as a fragrance widely used in pharmaceuticals, food products, and cosmetics, owing to its biological activities [36,37]. The other chemical marker of *T. nucifera* essential oil is 3-carene, a bioactive component that exhibits various biological activities, including antimicrobial, antioxidant, and fumigant properties [38,39]. However, further pharmacological and toxicological studies are required for practical application. As products of the isoprenoid pathway, 3-carene and limonene both originate from the common precursor geranyl pyrophosphate. The transcriptional regulation of terpene synthases is strongly influenced by environmental seasonality, resulting in adaptive shifts in the plant’s chemical phenotype [40].

As shown in Table 3 and Figure 2, seasonal and regional variations were observed in the targeted bioactive compounds, such as 3-carene and *D*-limonene, in *T. nucifera* oils during 2023–2024. According to the two-way ANOVA analysis, the concentration of *D*-limonene in *T. nucifera* essential oil was significantly more affected by season and the season × region interaction than that of 3-carene (Table 3). Similarly, the correlation matrix showed weak positive correlations between 3-carene and both temperature and rainfall, whereas *D*-limonene exhibited stronger correlations with rainfall and temperature (Table 4). Guimarães et al. [40] studied the influence of climatic parameters on the chemical profile of *Schinus terebinthifolia* leaf essential oils, which contained *D*-limonene (11.4–56.2%) and 3-carene (8.7–33.2%) as its major components. The chemical component of *D*-limonene showed a moderate positive and significant correlation with precipitation (*r* = 0.56, *p* < 0.05), while the component of 3-carene showed weaker correlations with the climatic parameters such as precipitation (*r* = 0.46). These results indicated that the concentration of *D*-limonene in *S. terebinthifolia* essential oil is more influenced by environmental conditions than that of δ-3-carene. Likewise, Cabrera et al. [41] found that *D*-limonene in essential oil from the leaves of *Myrocarpus frondosus* was directly proportional to environmental factors (temperature, solar radiation and rainfall) through multivariate analysis.

Seasonal variation had no significant effect on 3-carene levels in *T. nucifera* oils, whereas *D*-limonene concentrations were significantly influenced by season and season × region interaction from 2023 to 2024 (Table 3 and Figure 2). Regarding seasonal variation, 3-carene levels in *T. nucifera* oils remained consistent (8.7–30.8%) from 2023 to 2024, indicating no significant effect (Figure 2A). Guimarães, Silva, Andrade, Setzer, da Silva and Figueiredo [40] reported that the 3-carene content was relatively consistent (22.6 ± 7.1%), with no statistically significant difference between the dry and rainy seasons. Furthermore, the concentration of *D*-limonene in *T. nucifera* oils was significantly higher in summer and autumn compared to spring (Figure 2B). These findings were consistent with the results from other previous studies [42], which reported that the *D*-limonene content in oriental cedar oil was higher in summer (1.9 ± 0.0%) and decreased to 1.00 ± 0.1% in spring. Barra [43] emphasised that seasonal chemical variations are driven by multiple environmental factors, including precipitation, solar radiation, and temperature.

Regional variation significantly influenced 3-carene and *D*-limonene in *T. nucifera* oils (Table 3). These geographic differences in yield and chemical profiles of essential oils have been observed in several species, indicating that distinct chemotypes of plants grow in different locations [44,45]. For example, *Melaleuca alternifolia* essential oils exhibited notable differences in chemotypes depending on whether they originated from highland or coastal populations [46]. Similarly, *Origanum vulgare* from various Middle Eastern regions produced essential oils extracted from leaves that are dominated by either thymol or carvacrol, according to the region. Specifically, Saudi samples were chemotyped as carvacrol-type, while the Jordanian samples were identified as thymol-type [47]. In particular, regional variation is linked to multiple factors, especially exogenous factors including but not limited to region, soil and climate, affecting plant metabolisms [43]. Hence, regional variation in *T. nucifera* oils would be associated with chemotypic variation among plant populations. However, a comprehensive study based on an extensive collection of *T. nucifera* from diverse locations is essential to clarify chemotypic variation within the species [48]. Therefore, the seasonal and regional variation in the targeted components of *T. nucifera* oils, particularly *D*-limonene and 3-carene, is likely attributable to a combination of influences, including microclimatic conditions, genetic variation, and chemotypic differences. Elucidating the variable factors affecting the chemical profile and quantity of essential oils is challenging, as the biosynthesis of secondary metabolites can be influenced by natural processes such as plant development, rainfall, seasonality, temperature, and other environmental factors that impact the concentration of active constituents [49]. Several studies have proposed differing hypotheses to explain fluctuations in the targeted components of essential oils, based on their observed results [50,51]. In general, differential gene expression across species and metabolic pathways elicits complex, specific plant responses to environmental stress, resulting in decreased concentrations of some secondary metabolites while increasing others [50]. Furthermore, no significant differences were observed in the chemical markers, 3-carene and *D*-limonene between 2023 and 2024 with respect to seasonal factors in this study. Nevertheless, seasonal fluctuations, which have become increasingly pronounced in recent years due to climate change, are known to influence both the qualitative and quantitative characteristics of essential oils [52]. From a commercial perspective, long-term investigations are therefore required to elucidate potential interannual variations and their implications under changing climatic conditions.

Producing high-quality *T. nucifera* oils may therefore involve targeting chemical markers (3-carene and *D*-limonene). Oils from Jeju contained higher levels of 3-carene regardless of season, while oils from Hwasun showed enhanced *D*-limonene content during summer and autumn. Considering both oil yield and targeted bioactive components, *T. nucifera* oils extracted from Hwasun in autumn exhibited the highest concentrations of *D*-limonene and 3-carene, ranging from 77% to 86% throughout the study period.

## 4. Materials and Methods

### 4.1. Chemicals and Reagents

The standards (limonene: Cat #. 62118, purity: 99% and 3-carene: Cat #.21986, purity: 98.5%) were purchased from Fluka (Buchs, Switzerland). C_7_–C_40_ saturated alkanes standard mix (Lot #LRAC3115) was sourced from Sigma-Aldrich, St Louis, MO, USA. All incidental chemicals and reagents used were of analytical grade.

### 4.2. Experimental Site of Plant Material and Climate Characteristics

**Figure 3:**
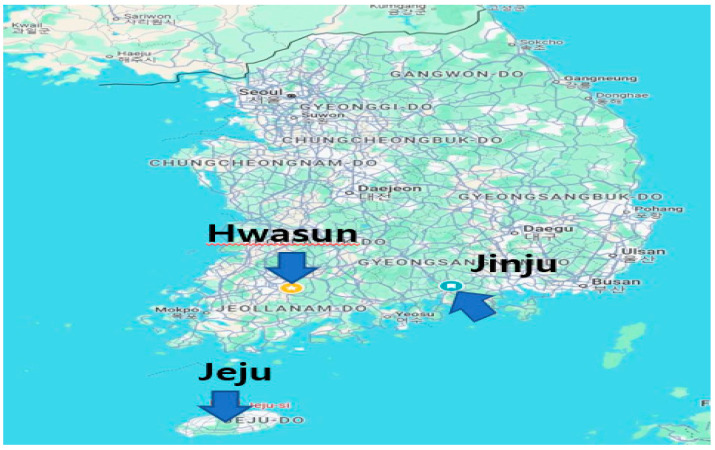
Geographical location of *T. nucifera* used in this study.

The fresh *T. nucifera* were collected from three different regions (Jeju, Jinju and Hwasun) in South Korea during spring (March), summer (June), and autumn (September) of 2023–2024 (see Figure 3).

**Figure 4:**
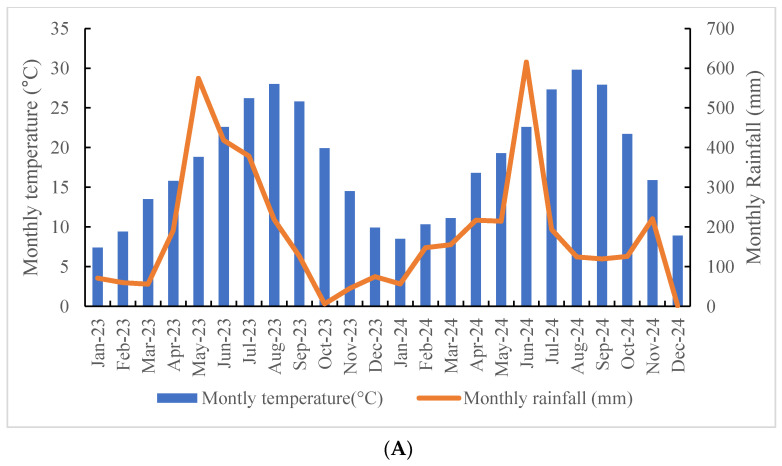

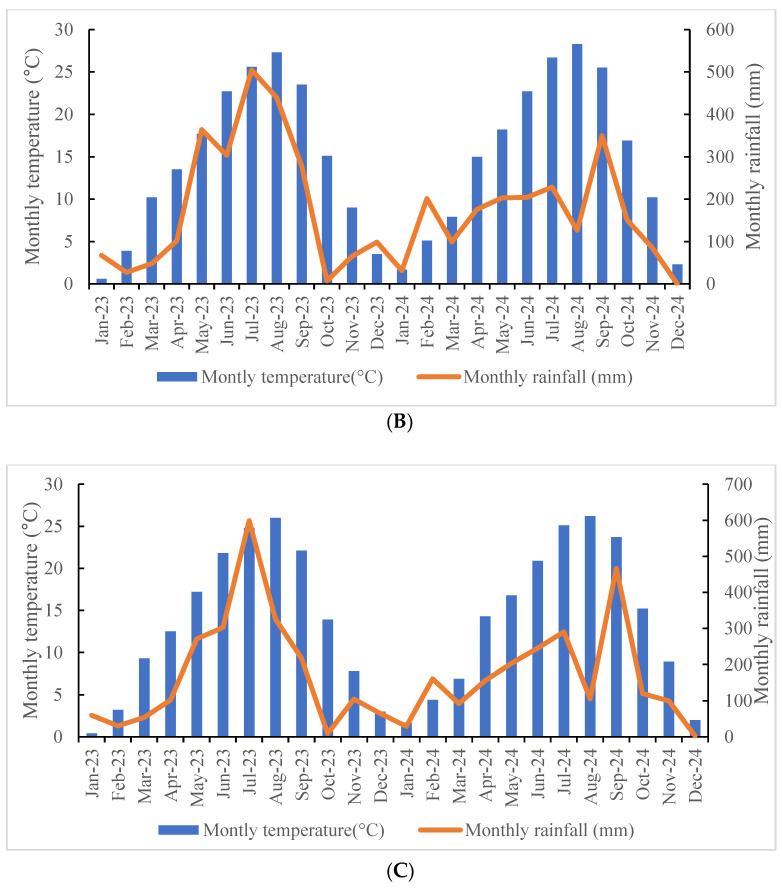
The monthly temperature (°C) and monthly rainfall (mm) of three different regions ((**A**): Jeju. (**B**): Jinju, (**C**): Hwasun) collected from *T. nucifera* from 2023 to 2024 (obtained from Korea Meteorological Administration National Climate Data Centre, https://data.kma.go.kr/cmmn/main.do, accessed on 30 March 2025). The first letter of the abbreviation represents the month, and the second to the year (2023–2024).

*T. nucifera* was identified, and voucher specimens were deposited at the herbarium (herbarium code: WFRC), Warm-Temperate and Subtropical Forest Research Centre at the National Institute of Forest Science (Jeju, South Korea). The climatic characteristics of each region were obtained from weather extrapolation (Figure 4). The database and tool are a comprehensive archive of climate data recorded by the Korea Meteorological Administration National Climate Data Centre [53].

The trial was conducted in a Randomised Complete Block Design (RCBD) of four blocks, and approximately 2 kg of *T. nucifera* samples were collected per individual tree in each block. The sampled plant was cut and labelled in the field before being stored on ice in a thermal-resistant container for transport to the laboratory. The samples were then weighed, and sub-samples were dried at 121 °C for 24 h to determine oven-dry weights (ODW). The remaining materials were packed in plastic bags and stored at −18 °C until extraction by hydrodistillation.

### 4.3. Hydrodistillation of Essential Oils

The essential oils were extracted from the leaves via hydrodistillation. Essential oils extraction was performed at the National Institute of Forest Science (Seoul, Republic of Korea). Each sample from each of the blocks at each seasonal time point and each region was divided into 2 × 1 kg duplicates. To minimise the variability during hydrodistillation, all samples were processed under identical conditions. Specifically, each sample (1 kg) was mixed with distilled water (DW) in a ratio of 1:7 (kg:L). Samples soaked in the DW were heated at 102 °C using a heating mantle (Model: MS-DM608, Serial number: 201602, Misung Scientific Co., Ltd.,Yangju, Republic of Korea). Then, the volatiles were condensed using a Dean-Stark trap. Extraction continued until no more essential oils were obtained. The yield of essential oils was calculated using the following equation.

Essential oil yield (%)=[essential oils distilled (g)/sample weight (ODWg)]×100%

The essential oils were dehydrated using an anhydrous sodium sulfate (98.5%, Samchun, Republic of Korea) and stored in a deep freezer until use.

### 4.4. Assessment of Aroma Characteristics in Essential Oils

The fragrance characteristics of the essential oil were analysed by a professional perfumer from Miscent (Seoul, Republic of Korea). Based on GC-MS data, not only the relative amounts of the volatile components but also their corresponding aroma descriptors were retrieved from the literature [22,23] and matched to the identified compounds. Specifically, fragrance descriptors commonly used for natural scents, such as herbaceous, camphorous, green, citrus, fruity, floral, woody, balsamic, aldehydic, and spicy, were used as reference categories. The intensities of the aroma attributes were scored on a scale from 0 to 5; the higher the score, the stronger the intensity, where 0 = none or not perceptible, 1 = very weak, 2 = weak, 3 = moderate, 4 = strong, and 5 = very strong. Each sample was evaluated three times by the professional perfumer on different days.

### 4.5. Analysis of Gas Chromatography-Mass Spectrometry (GC-MS)

The volatile constituents of the essential oils were analysed using GC–MS (7890B GC system coupled with a 5977A MSD, Agilent Technologies, Inc., Santa Clara, CA, USA) equipped with a VF-5MS capillary column (60 m × 0.25 mm, 0.25 µm; Agilent Technologies). The temperature of the GC injector was set to 280 °C, and the flow rate of the helium carrier gas was 2.0 mL/min. The initial oven temperature was 50 °C (5 min), followed by a temperature increase to 65 °C (30 min) at 10 °C/min. Thereafter, the temperature was raised to 210 °C (10 min) at 5 °C/min and, finally, to 305 °C (5 min) at 20 °C/min. The MS was conducted in the electron ionisation mode. The ion source and interface temperature were set to 270 °C and 250 °C, respectively, and a mass range of 35–550 amu was recorded in the full scan mode. The Kovats retention index (KI) of the individual compounds was evaluated by comparing their relative retention times with those of an n-alkanes mixture (C8–C30, Sigma-Aldrich, St. Louis, MO, USA) in a VF-5MS column. The volatile constituents were identified by comparing their calculated KIs with the reported values (e.g., NIST Chemistry WebBook). Quantification of the chemical markers was performed using the external standard method. Calibration curves for *D*-limonene and 3-carene were constructed using six concentration levels (200–1200 ppm). Each standard solution was injected in triplicate, and the peak areas were plotted against the corresponding concentrations to obtain linear regression equations. The calibration curves showed excellent linearity, with correlation coefficients (R^2^) of 0.996 and 0.997 for *D*-limonene and 3-carene, respectively. Quantification of these compounds in the essential oil samples was conducted based on their respective calibration equations. The remaining volatile components were expressed as relative percentage areas, calculated from the ratio of the individual peak area to the total peak area of all identified constituents, providing a semi-quantitative chemical profile of the essential oils.

### 4.6. Statistical Analysis

Data is presented as means ± SD (*n*  =  4). Statistical analyses were performed using SAS (Version 9.4, SAS Institute INC., Cary, NC, USA) software. The oil yield (% DW) and the concentration of 3-carene and *D*-limonene (%) in *T. nucifera* oil were subject to two-way (season × region) ANOVA followed by post hoc Tukey’s multiple range tests. Lastly, PCA was performed to examine the relationships among monthly temperature (°C), monthly rainfall (mm) and targeted chemical markers (3-carene and *D*-limonene, respectively).

## 5. Conclusions

*T. nucifera* essential oils are extracted from various parts, such as seeds, wood, and leaves. Historically, seed oils from *T. nucifera* have been used in food and hair care products, while the essential oils from its leaves remain largely unexplored regarding their extraction, chemical profile, bioactivity, and potential for commercial production. This study was the first to investigate how seasonal and regional factors affect the yield and chemical markers (3-carene and *D*-limonene) of *T. nucifera* leaf essential oils in South Korea. Oil yields remained stable across seasons (0.9 ± 0.2–1.6 ± 0.3%) but differed significantly by region (*p* < 0.05), with the highest yields observed in Hwasun. The consistent oil yield of *T. nucifera* likely results from the stable ecological conditions experienced by this evergreen, perennial species. A total of 36 volatile components were identified in the oils, primarily consisting of monoterpenes (83.7–90.4%) and sesquiterpenes (5.4–11.7%). The major components were α-pinene, 3-carene, and *D*-limonene, which together made up 79.4–86.6% of the total oils. *T. nucifera oils* exhibit diverse biological activities, primarily due to 3-carene and *D*-limonene, which were selected as chemical markers to elucidate seasonal and regional effects in this study. Regional factors significantly influenced 3-carene levels (*p* < 0.0001), while D-limonene was affected by season, region, and their interaction (*p* < 0.001), peaking in Hwasun during summer and autumn (up to 70.6%). For commercial production, high-quality *T. nucifera* essential oil can be optimised by targeting key markers, with Jeju oils showing higher 3-carene regardless of season and Hwasun oils exhibiting enhanced *D*-limonene content during summer and autumn. Therefore, *T. nucifera* essential oil from Hwasun harvested in autumn can be optimised for commercial production by maximising oil yield and enhancing chemical markers.

## Figures and Tables

**Table 1:** Leaf essential oil yield of *T. nucifera* collected from three regions (Jeju, Jinju, and Hwasun) and seasons (spring, summer and autumn) during 2023–2024.

	Essential Oils Yield (ODW, %)								
	Jeju			Jinju			Hwasun		
Season	2023	2024	Average	2023	2024	Average	2023	2024	Average
Spring	1.1 ± 0.2 ^y^	1.1 ± 0.2 ^y^	1.1 ± 0.2	1.2 ± 0.2 ^y^	0.9 ± 0.1 ^y^	1.1 ± 0.2	1.1 ± 0.1 ^x^	1.3 ± 0.1 ^x^	1.2 ± 0.1
Summer	1.2 ± 0.2 ^y^	1.2 ± 0.2 ^y^	1.2 ± 0.2	1.4 ± 0.3 ^y^	1.0± 0.1 ^y^	1.2 ± 0.3	1.4 ± 0.3 ^x^	1.6 ± 0.3 ^x^	1.5 ± 0.3
Autumn	0.9 ± 0.2 ^y^	1.5 ± 0.5 ^y^	1.2 ± 0.5	1.3 ± 0.1 ^y^	1.2 ± 0.3 ^y^	1.3 ± 0.2	1.4 ± 0.3 ^x^	1.6 ± 0.5 ^x^	1.5 ± 0.4
Essenti al oils yield: factor 1 (season): ns, factor 2 (region): * and factor 1 X factor 2 (interaction): NS									

Values are presented as means ± SD (*n* = 4). Mean values designated by a different letter are significantly different between groups (* *p* < 0.05, NS = not significant), according to two-way ANOVA test with season and region as variability factors: The effects of season and interaction (season and region) were not significant (ns and NS). Lower-case letters denote regional differences (x, y).

**Table 2:** Chemical components (%) in *T. nucifera* essential oils from different regions.

					Relative Area Percentage (%)		
	RT *	Compound	KI **	KI Ref ***	Jeju	Jinju	Hwasun
1	16.65	Tricyclene	919	927	0.1 ± 0.0	0.1 ± 0.0	0.1 ± 0.0
2	16.89	α-Thujene	921	930	0.1 ± 0.0	0.1 ± 0.0	0.1 ± 0.1
3	17.6	α-Pinene	927	931	22.6 ± 6.3	26.0 ± 0.1	33.0 ± 3.2
4	19.17	(+)-Camphene	941	946	0.7 ± 0.3	0.4 ± 0.2	0.6 ± 0.1
5	22.67	(-)-β-Pinene	972	980	0.8 ± 0.3	1.1 ± 0.0	1.2 ± 0.1
6	23.07	1-Octen-3-ol	976	981	0.1 ± 0.0	bdl	0.1 ± 0.0
7	24.28	β-Myrcene	987	991	1.6 ± 0.1	2.2 ± 0.2	1.8 ± 0.2
8	27.24	3-Carene	1009	1005	26.6 ± 5.2	12.6 ± 3.3	11.6 ± 3.1
9	31.2	D-Limonene	1032	1031	37.4 ± 8.4	40.9 ± 5.9	36.8 ± 5.8
10	37.43	β-cis-Ocimene	1042	1034	0.1 ± 0.0	0.1 ± 0.0	0.1 ± 0.0
11	44.64	3-Octyl_acetate	1126	1123	0.1 ± 0.0	bdl	0.1 ± 0.0
12	48.58	Terpinen-4-ol	1184	1177	0.1 ± 0.0	0.1 ± 0.0	0.1 ± 0.0
13	49.54	α-Terpineol	1198	1189	0.1 ± 0.0	0.1 ± 0.0	0.1 ± 0.0
14	51.54	Citronellol	1225	1228	0.1 ± 0.0	0.1 ± 0.0	0.1 ± 0.0
15	55.88	(-)-Bornyl_acetate	1284	1285	bdl	bdl	0.1 ± 0.0
16	60.07	δ-Eiemene	1346	1338	0.1 ± 0.0	0.3 ± 0.1	0.1 ± 0.0
17	65.27	β-Copaene	1439	1435	bdl	0.2 ± 0.0	0.1 ± 0.0
18	66.06	cis-β-Farnesene	1457	1455	0.1 ± 0.0	1.0 ± 0.2	0.3 ± 0.1
19	66.4	Humulene	1465	1457	bdl	0.5 ± 0.1	0.5 ± 0.2
20	67.14	γ-Muurolene	1483	1480	bdl	0.3 ± 0.1	0.1 ± 0.0
21	67.86	β-Cubebene	1500	1396	0.1 ± 0.0	0.4 ± 0.1	0.1 ± 0.0
22	68.25	γ-Cadinene	1512	1513	0.1 ± 0.0	0.9 ± 0.2	0.5 ± 0.1
23	68.74	δ-Cadinene	1527	1524	0.9 ± 0.3	3.6 ± 0.8	1.7 ± 0.1
24	68.91	trans-Calamenene_	1532	1529	0.1 ± 0.0	0.3 ± 0.1	0.2 ± 0.1
25	70.81	Spathulenol	1590	1591	bdl	bdl	0.1 ± 0.0
26	71.02	Gleenol	1597	1587	0.6 ± 0.2	0.7 ± 0.1	0.7 ± 0.1
27	72.3	α-Copaene	1627	1380	bdl	0.2 ± 0.0	0.1 ± 0.0
28	72.38	Epicubenol	1646	1629	0.1 ± 0.0	0.1 ± 0.0	0.1 ± 0.0
29	72.5	δ-Cedrol	1650	1645	2.2 ± 0.3	2.1 ± 0.2	2.1 ± 0.3
30	72.8	T-MuuroloI	1679	1657	0.3 ± 0.0	0.3 ± 0.1	0.3 ± 0.0
31	73.81	Levomenol	1698	1682	0.8 ± 0.1	0.7 ± 0.2	bdl
32	74.39	(Z, E)-Farnesol	1722	1722	bdl	0.1 ± 0.0	bdl
Monoterpene hydrocarbons					89.8 ± 1.5	83.5 ± 1.0	85.3 ± 1.2
Oxygenated monoterpenes					0.6 ± 0.1	0.2 ± 0.1	0.5 ± 0.1
Sesquiterpene hydrocarbons					1.4 ± 0.1	7.7 ± 1.0	3.5 ± 0.8
Oxygenated sesquiterpenes					4.0 ± 0.6	3.9 ± 0.5	3.2 ± 0.4
Total identified (%)					95.9 ± 2.0	95.4 ± 1.2	92.8 ± 1.5

RT *: Retention time (RT), KI **: Kovats Index (KI), KI ref *** (Kovats Index reference: NIST chemistry WebBook), bdl: below detection limit. Values are presented as means ± SD (*n* = 4).

**Table 3:** The chemical markers of 3-carene and *D*-limonene in *T. nucifera* essential oil (%) from two-way ANOVA with season, region and their interaction (season × region) as variability factors.

	The Chemical Markers of *T. nucifera* Essential Oil	
	3-Carene	*D*-Limonene
Season	NS	**
Region	***	**
Season × Region	NS	**

*** *p* < 0.0001, ** *p* < 0.001, NS = not significant (*p* > 0.05). and *n* = 4.

**Table 4:** Pearson correlation coefficients among monthly temperature (°C), monthly rainfall (mm), and chemical markers of 3-carene and *D*-limonene in *T. nucifera* essential oil.

	**3-Carene**	**Temperature**	**Rainfall**
3-Carene	1.000	0.1933	0.0099
Temperature	0.1933	1.000	0.571
Rainfall	0.0099	0.571	1.000
[3-carene] factor 1: temperature, factor 2: rainfall			
	*D*-Limonene	Temperature	Rainfall
*D*-Limonene	1.000	0.2125	0.3107
Temperature	0.2125	1.000	0.571
Rainfall	0.3107	0.571	1.000
[*D*-limonene] factor 1: rainfall, factor 2: temperature			

## Data Availability

The raw data supporting the conclusions of this article will be made available by the authors on request.
